# Evaluating the Effectiveness of Regional Ecological Civilization Policy: Evidence from Jiangsu Province, China

**DOI:** 10.3390/ijerph19010388

**Published:** 2021-12-30

**Authors:** Lingyun Mi, Tianwen Jia, Yang Yang, Lulu Jiang, Bangjun Wang, Tao Lv, Le Li, Junfeng Cao

**Affiliations:** School of Economics and Management, China University of Mining and Technology, Xuzhou 221116, China; jiatianwen123@cumt.edu.cn (T.J.); yangyang0202@cumt.edu.cn (Y.Y.); jianglulu@cumt.edu.cn (L.J.); taocumt@cumt.edu.cn (T.L.); TS20070348P21@cumt.edu.cn (L.L.); TS20070340P21@cumt.edu.cn (J.C.)

**Keywords:** ecological civilization, quantitative evaluation of policy text, ecological civilization evaluation index system, policy effectiveness

## Abstract

Evaluating the effectiveness of ecological civilization policies is the basis from which policymakers can optimize policies. From the perspective of the overall effectiveness of regional policies, and taking Jiangsu Province as an example, this study constructed a quantitative evaluation model of eco-civilization policy text and an eco-civilization evaluation index system. Using these tools, this paper evaluates the effectiveness of 53 ecological civilization policies issued by Jiangsu Province during 2004–2019 to promote the construction of ecological civilization in the four fields of resource utilization, environmental protection, economic development, and social life. There are three key findings. (1) During the period of 2004–2019, the effectiveness of the textual content of ecological civilization policies in Jiangsu Province generally showed a fluctuating upward trend. (2) The construction effectiveness indexes of the four fields of eco-civilization all showed a growth trend, but the construction effect varied greatly. The index of economic development had grown rapidly, while environmental protection had grown slowly. (3) Ecological civilization policies in Jiangsu Province were effective in promoting the construction of ecological civilization. However, the effects of different policy dimensions on ecological civilization development in the four fields were significantly different. Finally, based on these results, powerful recommendations are provided for the optimization of eco-civilization policies in Jiangsu Province. Moreover, Jiangsu is the first province in China to launch a provincial-level ecological civilization construction plan. Its policy optimization to promote ecological civilization construction can also provide an example and realistic basis for reference for the construction of eco-civilization in other provinces in China.

## 1. Introduction

China’s rapid economic growth has generated a series of environmental problems, such as water, soil and air pollution, energy shortages, and desertification [1,2,3,4]. The deterioration of the ecological environment has seriously affected China’s sustainable development [5] and even endangered human health [6,7]. To achieve its sustainable development goals and promote the coordinated development of its resources, environment, and social economy, it is imperative that China create “ecological civilization” [8,9], a concept first proposed at the 17th National Congress of the Communist Party of China (CPC). Ecological civilization refers to the total of material and spiritual achievements based on the protection and construction of a beautiful ecological environment. It is a form of human civilization. In 2018, China added “ecological civilization” to the constitution, giving it a higher legal status. With the advancement of eco-civilization construction, environmental, social responsibility and corporate governance (ESG) have become more familiar to people [10,11]. Promoting the recycling of resources through a circular economy, strengthening pollution control and ecological environmental protection, as well as promoting green production and lifestyle have all become important contents in the eco-civilization construction. Therefore, governments at all levels have issued relevant policies to promote the development of eco-civilization, and it has received widespread attention [12].

With the gradual enrichment of theory and practice, mature institutional design and clear institutional norms have increasingly become the key factors in constructing ecological progress [13]. However, studies have shown that despite the considerable number of policy documents issued by the government, the actual effect of China’s eco-civilization policies has not been as positive as expected [14]. Unfortunately, this issue has not yet attracted enough attention from scholars, for the following reasons. First, scholars have focused on evaluating the effects of constructing ecological progress [15,16,17], as well as on clustering [18], evolution [8], spatial local analysis [19], and coordinated development among fields [20,21]. However, they have not quantitatively evaluated the effects of policies targeting ecological construction, and the effectiveness of those policies has not been tested empirically. Second, research on eco-civilization policy is still at the stage of logical reasoning. Existing research has focused on the evolution and development of policies [21], and has proposed policy recommendations at the theoretical level [21,22]. However, there is a lack of quantitative analysis of policy texts, and policy evaluation lacks a quantitative basis. Finally, the eco-civilization policy is a complex system involving different construction fields. As a matter of fact, the government often promotes the construction of ecological civilization through multiple measures. The construction of ecological progress in a region faces the cumulative and comprehensive effects of many different types of policies initiated in different periods. Therefore, it is more consistent with the actual situation to study the effectiveness of policies from the perspective of overall policy efficacy.

To fill this gap, taking Jiangsu Province as an example, this study combined the effectiveness of policy text with the ecological construction index to assess the effectiveness of the policy. As China’s pioneer in reform and opening-up, and modernization, Jiangsu Province has actively explored the path of eco-civilization construction in recent years and proposed the first provincial-level ecological progress construction plan, which has been highly influential in other provinces. In addition, as the Chinese government issues ecological civilization policies, each provincial government will issue more targeted and specific policies according to the overall national guidelines and the actual situation of the province. Therefore, it is of more practical significance to evaluate the effectiveness of policies at the provincial level. At the same time, as the largest developing country, China’s policies and measures to promote the construction of eco-civilization not only provide reference experience for other developing countries in their ecological progress, but also make important contributions to global ecological and environmental governance, climate change mitigation, and balanced development of energy–economy–environment. This study aims to answer two questions that have not been addressed in existing studies: first, how does the effectiveness of eco-civilization policy text affect the four fields of ecological civilization construction (resource utilization, environmental protection, economic development, and social life)? Second, what are the effects of the four dimensions of policy text effectiveness (policy power, objectives, measures, and feedback) on promoting ecological civilization?

This research contributes to the literature in three ways: (1) it expands the study on policy antecedents of eco-civilization construction, establishes a quantitative evaluation model of the effectiveness of eco-civilization policy texts including policy power, policy objectives, policy measures, and policy feedback, as well as verifies the validity of the model, which provides a new perspective for the quantitative evaluation of policy texts. (2) It uses the entropy weight method to establish an index model for evaluating the effectiveness of provincial ecological civilization construction by screening indicators in four fields: resource utilization, environmental protection, economic development, and social life. This is an important supplement to the existing literature. (3) It incorporates the results of quantitative analysis of ecological civilization policy texts and the construction effectiveness index into the evaluation model together, evaluating the effectiveness of provincial ecological policies, which provides a new direction and path for the adjustment and optimization of regional eco-civilization construction policy.

## 2. Materials and Methods

### 2.1. Study Area

Jiangsu Province is located in the eastern part of mainland China, straddling the Yangtze River and the Huai River to the north and south, adjacent to the Yellow Sea to the east, Zhejiang Province to the south, Anhui Province to the west, and Shandong Province to the north, with a superior geographical location. At the same time, as one of the most economically developed provinces in eastern China, Jiangsu Province also plays an important position in the economy of the Yangtze River Delta. In recent years, Jiangsu Province has actively promoted the construction of ecological civilization and proposed the country’s first provincial-level ecological civilization construction plan, which has played a leading and exemplary role for other provinces. The reason for choosing Jiangsu Province is to judge whether the methods and viewpoints proposed in this study are helpful to the construction of eco-civilization and the optimization of eco-civilization policies.

### 2.2. Quantification of Ecological Civilization Policy Text

The ecological civilization that China is vigorously building is to seek the coordinated development of environmental protection, resource utilization, economic development, and social life. As Tkaczyński and Gacek stated in “China’s environmental policy in terms of European Union standard”, in recent years, the Chinese government has boldly introduced regulations that respect the environment, introduced policies to promote investment in low-carbon production, and advocated the ecological lifestyle. Policymakers issue more policies to promote ecological progress [23]. However, the quantitative analysis of ecological civilization policy is still in the initial stage, whereas the application of policy quantification in other fields is relatively mature. As early as 1978, Libecap [24] attempted to quantify regulations on mineral property rights in terms of the details of the content, the degree of accuracy, and similarities with earlier regulations. Murphy et al. [25] constructed a quantitative evaluation framework of energy policy for private residential buildings in the Netherlands based on the aspects of energy efficiency certificates, contracts, economic instruments, information tools, and building regulations. Peng et al. [26] established a three-dimensional quantitative model of policy power, policy objectives, and policy measures to analyze the collaborative evolution path of technological innovation policies. Later, Zhang [27] applied this three-dimensional model to analyze and evaluate the effectiveness of China’s energy conservation and emission reduction policies. Lan [28] also used this three-dimensional model to analyze and evaluate the effectiveness of Chinese government policies in promoting renewable energy development. Considering the important role of feedback in policy implementation, Mi et al. [29] introduced policy feedback based on the three-dimension model of policy power, policy objectives, and policy measures and established a four-dimension model to quantitatively evaluate the textual effectiveness and policy effect of China’s energy conservation policies. Mi’s four-dimensional model has been recognized by scholars. For example, Wang and Zhu [30] used the four-dimensional model to evaluate the policy efficacy and effect of China’s industry-university-research cooperative innovation, thus proving the validity of Mi’s four-dimensional model. Wu et al. [31] also applied this four-dimensional model to evaluate the effectiveness of policy documents and implementation effects in promoting hierarchical diagnosis and treatment in China. The validity of the four-dimensional model is further demonstrated. Referring to the above methods, this study adopts the four-dimensional model of policy power, objectives, measures, and feedback to analyze ecological civilization policies. This can fully evaluate the process of policy promulgation, implementation, and feedback, and the results comprehensively reflect the content validity of ecological civilization policies.

Our evaluation of policy effectiveness adopts the policy text analysis method. According to the content of the policy text, each dimension is given a score of 1–5. Referring to the regulations of the State Council on procedures for formulating rules [32], and the evaluation methods used in Zhang et al. [27] and Shen et al. [33], policy power is mainly scored according to the policy promulgation institution and policy type. Policy objectives are divided into resource utilization, environmental protection, economic development, and social life based on the ecological civilization policy promulgated by Jiangsu Province and the “Overall Plan for the Reform of the Ecological Civilization System” issued by the Chinese government. The values are then assigned according to the attitude intensity of the construction objectives in the four fields and the detailed degree of the policy. Policy measures are divided into three types: command-control, economic incentive, and information participation according to Murphy et al. [25] and Mi et al. [29] and are scored according to the degree of clarity, enablement, and enforcement of the policy content. Policy feedback is scored according to the supervision mode, supervision department, and feedback mechanism of the policy according to Mi et al. [29]. The quantitative criteria for policy text are shown in Table A1 of the Appendix A.

Prescoring was adopted to narrow the subjective differences among raters and maintain the unity of opinions. Fifteen articles from 53 policy texts were randomly selected, three group raters scored them independently, and then the fourth group checked and compared the results of each group. For inconsistent information, the consensus was reached through backtracking and discussion. Finally, the scoring of all policy texts was completed. Referring to studies of Peng et al. [26], Zhang [27], and Li et al. [34], the effectiveness quantification model in this study uses multiplication because the effectiveness of the policy text reflects the comprehensive effectiveness of each dimension of the policy. Due to the complexity of the ecological civilization system, the four dimensions of policy power, policy objectives, policy measures, and policy feedback have different nature and meanings in different policies and different fields. Therefore, they cannot be simply added together. Using multiplication can more effectively reflect the synergistic effect of each dimension. The effectiveness quantification model is shown in Formula (1).

(1) $$PMG_{t}=\sum_{i=1}^{N}p_{i}*m_{i}*c_{i}*f_{i}$$

In Formula (1), *t* is the year when the policy was issued; *N* is the number of policies promulgated in year *t*; *i* is the policy *i* implemented in year *t*; *p_i_*, *m_i_*, *c_i_*, *f_i_* are the policy power, policy objectives, policy measures, and policy feedback of policy *i*; *PMGt* is the total effectiveness of the ecological civilization policy text in year *t*.

### 2.3. Construction of an Ecological Civilization Evaluation Index System

The effectiveness assessment of ecological civilization policy needs to be based on the actual effects of ecological civilization construction. Ecological civilization is a complex system intertwined with the environment, economy, society, and resources, so its evaluation system also needs to take into account the different standards of each system. At present, scholars have established systems at different levels to evaluate the effects of ecological civilization construction. At the national level, Zhang et al. [35] constructed an ecological civilization evaluation system based on four aspects: green environment, green production, green living, and green infrastructure. At the provincial level, Chai et al. [36] constructed an indicator system based on the three subsystems of economy, society, and nature to measure and rank the optimal indices of ecological civilization construction in China’s provinces. Dong et al. [37] used geographically temporally weighted regression (GTWR) to analyze the determinants of ecological civilization performance of Chinese provinces from a “five-in-one” perspective. At the urban level, Yu et al. [38] constructed an evaluation index system based on the sustainable development of urban space in three aspects: green coordination, green development, and green sustainability. Meng et al. [39] established a composite indicator of ecological development to measure the relationship between urban ecological civilization and economic development. These studies focused on evaluating the effect of ecological civilization construction, and the classification criteria of each evaluation system vary greatly, leading to major differences in evaluation results. Therefore, to evaluate the effectiveness of ecological civilization policies, it is necessary to establish an ecological civilization evaluation index system based on policy objectives.

First, based on the definition and overall direction of ecological civilization, and the policy text analysis results, this study extracted the policy objectives and divided ecological civilization construction into four fields: resource utilization, environmental protection, economic development, and social life. Second, the index database was established based on the “assessment target system of ecological civilization construction” [40] issued by the National Development and Reform Commission of China, “measures for the evaluation and assessment of ecological civilization construction objectives of Jiangsu Province” [41] formulated by the Jiangsu provincial government, and the existing achievements of the ecological civilization evaluation system. Then, according to the representativeness of indicators and the availability of data, an ecological civilization evaluation system including 28 specific indicators was constructed. Finally, the index weight design was carried out. The results are shown in Table 1.

Table 1 shows that the selection of indicators in the resource utilization field mainly focuses on resources utilization in industry, agriculture, and daily life, such as industrial solid waste utilization, per capita agricultural fertilizer application, and harmless treatment rate of domestic garbage. Indicators in the field of environmental protection focus not only on environmental resource protection and pollution discharge but also on pollution control, such as the green coverage rate, per capita wastewater discharge, and industrial pollution control investment. Indicators in the field of economic development not only focus on the economic aggregate but also measure the economic structure and economic development, such as per capita GDP, the proportion of tertiary industry in GDP, and technical market turnover. In people’s daily life, housing and transportation are important issues that affect people’s livelihood at this stage. In addition, medical services and education levels are important indicators reflecting people’s social life. Therefore, indicators in the field of social life mainly include per capita housing area, per capita urban road area, number of physicians per 10,000 people, and number of college students in 10,000 persons.

Due to the differences in character, positive and negative orientations, and measurement units of each index, dimensionless processing is needed before data analysis. Because the index has zero value after dimensionless processing, to facilitate the weight design in the next step, the forward index and the reverse index were processed using Formulas (2) and (3):

(2) $$x*=\frac{x_{ij}-x_{j\min}}{x_{j\max}-x_{j\min}}+1$$

(3) $$x*=\frac{x_{j\max}-x_{ij}}{x_{j\max}-x_{j\min}}+1$$

In Formulas (2) and (3), x* is the positive value; *x_ij_* is the sample *i* of the index *j*; *x_j_*_max_, *x_j_*_min_ represent the maximum and minimum sample values *j*, respectively.

Index weight assignment is the key link of ecological civilization effect evaluation. The commonly used weight assignment methods mainly include subjective weighting and objective weighting. Subjective weighting is easily affected by the experts’ subjective consciousness and has a certain deviation, which cannot correctly reflect the relationship between the indicator data. The entropy weight method is an objective evaluation method that can be used for multiple objects and multiple indicators. According to the variation degree of each indicator, the entropy weight of each indicator is calculated by using the information entropy, and then the weight of each indicator is modified by the entropy weight, to obtain the objective index weight. The evaluation results are mainly based on objective data, which are almost unaffected by subjective factors, and can largely avoid human factors interference. Because the ecological civilization evaluation system involves different fields, and there are many indicators with different meanings, it is more reasonable to use the objective weight assignment method. Therefore, this study referred to Li and Li [42] and He et al. [43], who used the entropy weight method to determine the weights of each index.

To evaluate the level of ecological civilization construction in year *m*, the system includes *n* indicators, the original indicator data matrix is established (Formula (4)), and then the information entropy *Y* is calculated (Formula (5)). *p_ij_* is the probability that the system is in the corresponding state, and *k* is a constant that is related to *m* (*i* = 1, 2, …, *m*; *j* = 1, 2, …, *n*).

(4) $$X={\left(x_{ij}\right)}_{m\times n}$$

(5) $$Y=-k\sum_{i=1}^{m}p_{ij}\ln p_{ij}$$

Specific steps are as follows:

(1) Calculate *p_ij_* after dimensionless processing:

(6) $$p_{ij}=x_{ij}/\sum_{i=1}^{m}x_{ij}$$

(2) Calculate the entropy value *e_j_*:

(7) $$\begin{array}{c} e_{j}=-k\sum_{i=1}^{m}p_{ij}\ln p_{ij}\\ \left(k=1/\ln m\right) \end{array}$$

(3) Calculate the difference coefficient of indicators *d_j_*:

(8) $$d_{j}=1-e_{j}$$

(4) Calculate the weight of the index *w_j_*:

(9) $$w_{j}=d_{j}/\sum_{j=1}^{n}d_{j}$$

(5) Calculate the effectiveness index of ecological civilization *U_i_*, in which a greater value of *U_i_* corresponds to higher effectiveness of the ecological civilization construction:

(10) $$U_{i}=\sum_{j=1}^{n}w_{j}p_{ij}$$

### 2.4. Effectiveness Evaluation Model of Ecological Civilization Policy

To evaluate the effectiveness of ecological civilization policies in different fields, a regression model was established. The model used the text effectiveness of the four dimensions of ecological civilization policies as independent variables and the effectiveness index of ecological civilization construction as dependent variable. The regression analysis has been widely used to test the effects of policies in economic development [44], technological innovation [45,46], and energy conservation and emissions reduction [47,48]. Because the construction effect of the previous year has a significant impact on ecological civilization construction this year, the ecological civilization construction index of the previous year is considered in the regression model. Considering that in real life, most policies are issued at different times of the year, and the effectiveness of the policy takes some time to appear, the policy’s lagging effect was introduced to the formula. This approach can also reduce potential endogeneity problems and has been used in many studies, such as those of Costantini et al. [49] and Schleich et al. [50]. In addition, to stabilize the data, all variables except the model constants and the random influencing factor variables adopted natural logarithms [51]. The policy effectiveness evaluation model is shown in Formula (11).

(11) $$\begin{array}{c} \ln U_{t}=C_{t}+\alpha \ln PU_{t}+\beta_{1}\ln p_{t-i}+\beta_{2}\ln m_{t-i}^{\varphi }+\beta_{3}\ln c_{t-i}^{\delta }+\beta_{4}\ln f_{t-i}+\varepsilon_{t}\\ t\in [2004,2019] \end{array}$$

The definitions of variables in Formula (11) are shown in Table 2:

### 2.5. Data Source

The ecological civilization policy of Jiangsu Province was searched for in Wan fang, Peking University’s magic weapon, global law and regulations network, and the Jiangsu provincial government’s official website with the keywords “ecological civilization” and “ecological civilization construction”. As the Chinese government issues ecological civilization policies, the Jiangsu provincial government will issue more targeted and specific policies according to the overall national guidelines and the actual situation of the province. For example, after the announcement by the national government departments of the “Eleventh Five-Year Plan for National Ecological Protection” and the “Twelfth Five-Year Plan for National Ecological Protection”, Jiangsu Province issued the “Eleventh Five-Year Plan for Environmental Protection and Ecological Construction in Jiangsu Province” and the “Twelfth Five-Year Plan for Environmental Protection and Ecological Construction in Jiangsu Province”. In this way, the country’s goal of ecological civilization construction has led to the detailed rules of the ecological civilization policy of Jiangsu Province. Therefore, to prevent double-counting of policies between the state and Jiangsu Province, this study only selected Jiangsu Province policy for quantitative analysis. Ultimately, 53 policies were selected. The policy issuance agencies include the government of Jiangsu Province, the environmental protection department, the forestry bureau, and the department of land and resources of Jiangsu Province (53 policies are shown in Supplementary Table S1).

The data of each indicator in the ecological civilization evaluation index system are derived from the Jiangsu Statistical Yearbook [52] and National Bureau of Statistics of the People’s Republic of China [53]. Considering that ecological civilization policies in Jiangsu Province first appeared in 2004, and the availability of indicator data, this study took 2004 as the start of the period in which to evaluate and analyze the policy texts and policy effectiveness. The full study period is from 2004 to 2019.

## 3. Results

### 3.1. Quantitative Results of Ecological Civilization Policy Text

**Figure 1:**
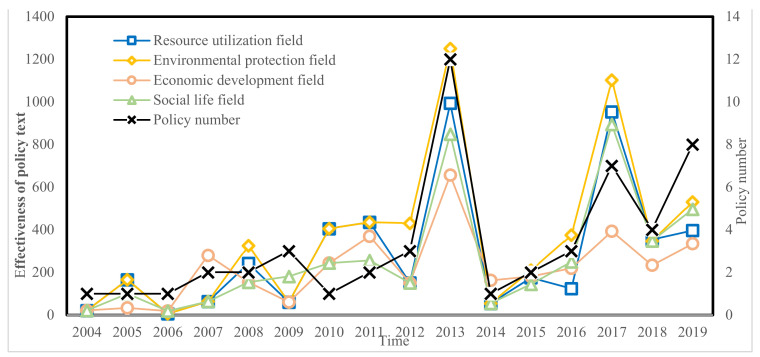
Effectiveness of the ecological civilization policy text from 2004 to 2019.

After sorting out the policy texts on the construction of eco-civilization in Jiangsu Province from 2004 to 2019, we conducted a quantitative evaluation of the policies promulgated based on the quantitative evaluation model of eco-civilization policies established in Section 2.2. The quantitative results of the ecological civilization policy text of Jiangsu Province are shown in Figure 1. The policy text effectiveness values of the four fields of ecological civilization construction in Jiangsu Province show an increasing trend. Specifically, before 2012, the effectiveness value of policy text in the four fields increased steadily. After 2012, the growth rate was significantly improved, with the largest increases in 2012–2013 and 2016–2017, and the total effectiveness value of policy text in the four fields reached its peak in 2013. There are two main reasons for this: one is mainly because after the state formulated ecological civilization policies in the “Twelfth Five-Year Plan” (2011–2015), the Jiangsu Provincial government also issued related policies such as the “Jiangsu Province Ecological Civilization Construction Plan (2013–2022)” and the “Twelfth Five-Year Plan for Environmental Protection and Ecological Construction in Jiangsu Province”; another is because, with the release of the “Opinions on Accelerating the Construction of Ecological Civilization” by the CPC Central Committee and The State Council, Jiangsu Province also issued the “Implementation Opinions of Jiangsu Provincial Party Committee and Provincial Government on Accelerating the Construction of Ecological Civilization”, clarifying the objectives and tasks of the construction of ecological civilization, to significantly improve the effectiveness of the policy.

In different years, the effectiveness of ecological civilization policy text shows large fluctuations. In particular, from 2013 to 2017, policy effectiveness is U-shaped in the four fields. Moreover, there is a large difference in policy text effectiveness among the four fields, with the policy text effectiveness in the field of environmental protection being relatively high, and that in the economic development field being relatively low. In addition, the trend of policy text effectiveness is similar to that of policy quantity, which indicates that the improvement of policy text effectiveness is mainly driven by the cumulative number of policies. The textual effect of a single policy makes an insufficient contribution to the overall effectiveness of the policy.

### 3.2. Analysis Results of Ecological Civilization Construction Effect

**Figure 2:**
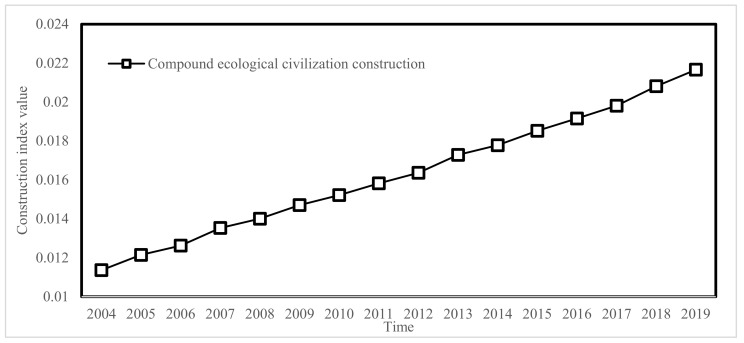
Compound ecological civilization construction index from 2004 to 2019.

**Figure 3:**
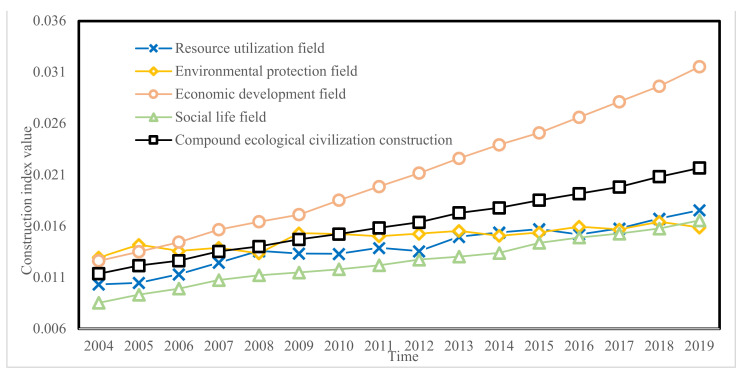
Ecological civilization construction index in four fields from 2004 to 2019.

According to the evaluation model of the eco-civilization construction effectiveness index established in Section 2.3, we evaluated 28 eco-civilization construction indexes selected from 2004 to 2019 in Jiangsu Province, as shown in Table 3, Figure 2 and Figure 3. From 2004 to 2019, the ecological civilization construction index of the four fields shows an overall growth trend, indicating that ecological civilization construction in Jiangsu Province has achieved remarkable results. However, construction effects in different fields are quite different, and coordinated development has not been achieved. The growth range in the economic development field is the largest, with an extreme difference of 0.0189 over 16 years, 2.5 times higher than in 2004; followed by the social life field, where the growth rate is stable. The index of the resource utilization field shows small negative growth in 2009, 2010, 2012, and 2016. The index in the field of environmental protection shows the slowest growth, and even negative growth in 2006, 2008, 2010, 2011, 2014, and 2017. This indicates that Jiangsu Province has developed rapidly in the fields of economic development and social life, and needs further improvement in the resource utilization and environmental protection fields.

### 3.3. Effectiveness Evaluation Results of Ecological Civilization Policy

To test the effectiveness of eco-civilization policies in different fields, we put the quantitative analysis results of policy texts and evaluation index of ecological civilization construction into the model, and a regression analysis was conducted for the four fields. The results are shown in Table 4. The results show that R^2^ is greater than 90%, and both the F value and Durbin–Watson statistic are within the acceptable range, indicating a good fit effect in the four models. The VIF values of the variables in each field are all less than 10, indicating that there are no multiple mutual linear problems. According to the AIC and SC quasi-test, when the two values are the minimum, the optimal lag length is determined. Therefore, the lag time of each policy dimension was determined to be within 3 years, which indicates that the estimated results of the model can explain the actual situation well.

Table 4 shows that in the resource utilization field, policy feedback has significant effects. Whereas policy power and policy objective effect are not significant. Among the policy measures, economic incentive policies have a significant effect on the construction of ecological civilization, and command-control policies and information participation policies have a negative effect.

In the field of environmental protection, both policy power and policy feedback show significant effectiveness, and the effect of policy feedback is greater than that of policy power. However, policy objectives have a negative disincentive effect. Among the three types of policy measures, both economic incentive policies and information participation policies have insignificant effects, and command-control policies have a negative disincentive effect.

In the economic development field, policy objectives have shown remarkable effectiveness, while the effect of policy power and policy feedback is not significant. Among the policy measures, information participation policies have no significant effects, while command-control policies and economic incentive policies show negative effects.

In the field of social life, policy power shows significant effectiveness, while both policy objectives and policy feedback do not show significant effectiveness. Among the policy measures, economic incentive policies have a positive effect on social life, information participation policies have no significant effect, and command-control policies have a negative effect. In the four fields, the achievements of ecological civilization construction in the previous year have the most significant effect on the ecological civilization construction in the following year.

## 4. Discussion

### 4.1. Analysis on the Evolution of Policy Effectiveness from 2004 to 2019

(1) During 2004–2019, the overall effectiveness of the ecological civilization policy texts in Jiangsu Province is increasing, which shows that the Jiangsu Provincial government attaches increasing importance to the development of ecological civilization and has issued a considerable number of policy documents to promote the ecological civilization construction. However, the value of policy effectiveness fluctuates greatly from year to year, which is related to the fact that the government’s policy-making efforts are focused on short-term goals and lack strategy. Moreover, changes in the effectiveness of ecological civilization policies are mainly caused by policy numbers, and the textual effect of a single policy makes an insufficient contribution to the overall effectiveness of the policy. This is a common problem in government policy-making. The evaluation results of Mi et al. [29] on energy conservation and emission reduction policies and the evaluation results of Wang and Zhu [30] on industry-university-research collaborative innovation policies all reflect that the effectiveness of a single policy is insufficient and the policy quality needs to be improved.
(2) The overall effectiveness of ecological civilization construction in Jiangsu Province is remarkable and shows an overall upward trend. However, the construction effects in four different fields vary greatly. The fields of economic development and social life have developed rapidly, while negative growth is still observed in the resource utilization field and environmental protection field. The overall ecological civilization construction has not yet achieved coordinated development. This finding is consistent with the results of Wang and Chen [17] on the effectiveness of China’s ecological civilization. They found that the development level of ecological civilization in Jiangsu Province showed an upward trend, and the ecological environment development lagged behind the economic and social development. Environmental quality is the most important bottleneck restricting the development of future ecological civilization [35,54]. Wang et al. [55] also pointed out that the “pollute first, govern later” in Jiangsu Province has not yet been fundamentally reversed. Additionally, this study also finds that there is a significant deviation between policy input preferences and ecological civilization construction effects. The government issued the most policies in the field of environmental protection, followed by resource utilization, and the policy texts in those fields were significantly more effective than those in the fields of economic development and social life. However, increased policy input is far less effective than expected in promoting resource utilization and environmental protection, which needs to be taken seriously by policymakers. To promote the coordinated development of the four fields of ecological civilization, policymakers need to flexibly apply different types of policy measures or policy tool combinations [56].

### 4.2. Analysis of Resource Utilization Fields

In the field of resource utilization, only policy feedback has a significant promoting effect, with neither policy power nor policy objectives effects reaching significance levels. Among the policy measures, the economic incentive policy has a significant effect on the construction of eco-civilization in the resource utilization field, while the command-control policy and the information participation policy are not significant. As an important field of ecological environment assessment, resource utilization is the core content of promoting eco-civilization construction [57,58]. However, Jiangsu Province is a large economic province with many energy-intensive and resource-intensive enterprises. It is inevitable to face many difficulties to improve resource utilization and promote ecological construction in the field of resource utilization. Therefore, the eco-civilization policy documents promulgated by Jiangsu Province mainly focus on economic incentive policies, which promote technological innovation of enterprises to improve resource utilization efficiency through financial investment, while constantly improving and perfecting the supervision and feedback mechanism. This has led to the effectiveness of policy feedback and economic incentive policy measures. However, most of the existing policy documents to promote resource utilization are notices, which are not legally binding and have weak administrative binding force. Moreover, there is a lack of clear goals for resource utilization efficiency in notification policies. Thus, the policy power and policy objectives do not have significant effectiveness in the field of resource utilization.

Therefore, in the field of resource utilization, it is necessary to improve the legal force of policy promulgation and strengthen the effect of policy power and policy objectives on promoting resource utilization. The government should also attach importance to command-control policy and the information participation policy, and strengthen the promotion and implementation of eco-civilization policies based on realistic characteristics. At the same time, the government should establish a sound feedback and supervision system to ensure more timely policy feedback and more accurate adjustments.

### 4.3. Analysis of Environmental Protection Fields

In the field of environmental protection, policy power and policy feedback can positively promote the construction of ecological civilization, while policy objectives and policy measures have no significant effect. Environmental protection is an important part of ecological progress [59]. As a pioneering demonstration area for ecological improvement, Jiangsu Province is strengthening the environmental inspection system while also constantly improving the public participation and supervision mechanism of environmental assessment. At the same time, with the increasing investment in environmental protection and ecological restoration, the public is more aware of the benefits of ecological environment improvement [60]. This sense of gain encourages more people to actively participate in the supervision and feedback of the implementation of environmental protection policies. Therefore, policy power and policy feedback have shown significant effectiveness in promoting eco-civilization construction in the field of environmental protection. However, there is a certain conflict between economic development and environmental protection [35]. How to strengthen the synergistic promotion effect of policy objectives in economic development and environmental protection must be taken seriously [61]. Moreover, in the field of environmental protection, in addition to formulating more detailed and quantifiable policy objectives, a detailed eco-civilization assessment system and abundant ways of public participation are also crucial.

Therefore, in the field of environmental protection, it is necessary to improve the degree of coordination between environmental protection and resource utilization when formulating policy objectives. Reducing resource waste by improving the resource utilization rate can not only promote environmental protection but also improve the sustainability of the economy. In addition, the pressure function of the command-control policy and the dynamic function of the economic incentive policy should be given full play. While improving its compulsory driving force, the government should also pay attention to using economic means to guide enterprises and the public to take the initiative to protect the environment.

### 4.4. Analysis of Economic Development Fields

In the field of economic development, policy objectives have shown significant effectiveness, but policy power, policy feedback, and policy measures have not reached a significant level. Economic development targets have been receiving more attention as the focus of local government performance reviews [62,63]. For example, Du et al. [63] argue that the key to coordinated development is to achieve a win–win situation between economic development and ecological protection. Peng et al. [64] regarded economic quality as the main factor affecting the construction of ecological civilization in Jiangsu Province. However, there are some conflicts between environmental protection and economic growth in the short term [35]. When policy power is strengthened in environmental protection supervision, it will inevitably lead to economic constraints for some enterprises with insufficient environmental protection capabilities. Moreover, in the ecological civilization policy issued by Jiangsu Province, environmental protection has higher privilege than economic development does. Although there are relatively clear development goals in the field of economic development, relatively few policy measures and various indicators are specified, which is not conducive to direct public participation in supervision and feedback. This may be the reason why the policy objectives are more significantly driven, but the policy power, policy feedback, and policy measures are not significant for the economic development field.

Therefore, in the field of economic development, the government should improve policy power, feedback, and objectives, and promote the implementation of command-control policy, economic incentive policies, and information participation policies. In addition, it should enrich the content and form of information participation policies, and establish the correlation between the public and the results of sustainable economic construction, thus increasing the public’s enthusiasm about promoting economic development through self-association and self-perception.

### 4.5. Analysis of Social Life Fields

In the field of social life, policy power shows a significant promoting effect, while the effect of policy objectives and feedback is not significant. Among the policy measures, economic incentive policies show significant effectiveness, with neither command-control nor information participation policies having a significant promotional effect. The improvement of social life involves all aspects of people’s lives, and it is also closely related to economic development, environmental protection, and resource utilization. Most of the policy objectives involved are relatively macro or broad, which is not conducive to the direct participation of social organizations as well as the public in supervision and feedback. This could be the reason why the effect of policy objectives and policy feedback is not significant. Economic incentive policies are invested in all aspects of life in the form of financial subsidies. These direct economic inputs can make the public feel the effects of input and output. Command-control policies reflect the constraints of compulsory administrative power and lack the motivation of subjective initiative of actors [29]. Once the supervision in policy implementation is not in place, the policy effect will be greatly reduced. However, most of the information participation policies that have been published are advocating, lacking specific implementation plans and action guidance. The way of information participation is also one-way communication, mainly publicity and education. Therefore, command-control and information participation policies are not effective in promoting social life fields.

Yan et al. [65] believe that to accelerate the construction of ecological civilization, it is necessary to promote the coordination between social harmony and human–environment harmony. Therefore, in the field of social life, the government should pay attention to the harmonious development of ecology and society, while improving policy objectives, economic incentive policies, information participation policies, and policy feedback effectiveness. On the one hand, the policymakers need to refine policy objectives, promulgate policy documents with clear development objectives, vigorously implement economic incentives and information participation policies, while increasing financial investment and improving the construction of public service institutions. On the other hand, they can strengthen the supervision and feedback mechanism, identify the most pressing problems from the supervision department and the public, and optimize policies to improve public life.

## 5. Conclusions

To evaluate the effectiveness of regional eco-civilization construction policies, this study expanded the policy antecedent research of eco-civilization construction, and constructed a quantitative evaluation model of eco-civilization policies from the four dimensions of policy power, policy objectives, policy measures, and policy feedback, providing a new perspective for conducting the quantitative evaluation of policy texts. Then, taking Jiangsu Province as an example, the entropy weight method was used to measure the eco-civilization index, and an evaluation model of the provincial eco-civilization effectiveness index was established. After that, the quantitative analysis results of the ecological civilization policy text and the construction effect index were incorporated into the evaluation model to evaluate the 53 eco-civilization policies promulgated in Jiangsu Province during 2004–2019 in the fields of resource utilization, environmental protection, economic development, and social life. The results showed that during 2004–2019, the effectiveness of Jiangsu Province’s ecological civilization policy showed a fluctuating upward trend. However, the effectiveness index of eco-civilization construction varied widely in the four fields of resource utilization, environmental protection, economic development, and social life. Among them, the index of economic development increased rapidly, while the index of environmental protection increased slowly. Although eco-civilization policies of Jiangsu Province show effectiveness in promoting ecological civilization, there are significant differences in the effects of different policy effectiveness dimensions on the four fields of eco-civilization construction. Policymakers need to coordinate the optimization of policy tools according to the policy objectives in different fields. These results not only provide direction and basis for the optimization of eco-civilization policy in Jiangsu Province but also provide an example and reference realistic basis for other Chinese provinces and developing countries to promote eco-civilization construction.

Although our study provides a theoretical basis for the optimization of provincial ecological civilization policies, there are some limitations. First, due to China’s vast territory, there are significant differences in the natural environment, resource endowments, and economic and social development levels among the eastern, central, and western provinces. Jiangsu was the first province to propose the construction of ecological civilization at the provincial level, which serves as an example and model for other provinces. However, when it is extended to other provinces, policymakers need to design and optimize policies according to local conditions. In future research, we can consider the evaluation and comparison of eco-civilization construction across provinces. Second, when evaluating the effectiveness of policy texts, it relies on expert scoring. Experts’ interpretation of the evaluation criteria is somewhat subjective. Delphi Method can be considered to improve the effectiveness of expert scoring in future research. Finally, in the selection of the indicator system for the effectiveness of eco-civilization construction in the four fields, only some indicators were selected due to the limitation of data availability and representativeness. In the future, with sufficient data, more diversified indicators can be considered to evaluate the effectiveness of eco-civilization construction in a more comprehensive way.

## Figures and Tables

**Table 1:** Ecological civilization effectiveness evaluation index.

Target Layer	Criterion Layer	Index Layer	Unit	Character	Weight
Evaluation index system of ecological civilization	Resource utilization A 0.223	A1 Sewage treatment rate	%	Positive	0.030
		A2 Daily sewage treatment capacity of city	Million m^3^	Positive	0.030
		A3 Per capita daily water consumption	Liter	Negative	0.024
		A4 Industrial solid waste utilization	%	Positive	0.028
		A5 Harmless treatment rate of domestic garbage	%	Positive	0.033
		A6 Unit GDP power consumption	kW ⋅ h/10,000 yuan	Negative	0.038
		A7 Per capita agricultural fertilizer application	Ton/person	Negative	0.040
	Environmental protection B 0.239	B1 Per capita sulfur dioxide emissions	Kg/person	Negative	0.036
		B2 Forest coverage rate	%	Positive	0.060
		B3 Per capita wastewater discharge	Ton/person	Negative	0.037
		B4 Per capita park green area	m^3^	Positive	0.022
		B5 Nature reserve area	Million hectares	Positive	0.037
		B6 Industrial pollution control investment	10,000 yuan	Positive	0.031
		B7 Green coverage rate	%	Positive	0.016
	Economic development C 0.337	C1 Per capita GDP	Yuan	Positive	0.037
		C2 Proportion of tertiary industry in GDP	%	Positive	0.038
		C3 Per capita disposable income of urban households	Yuan	Positive	0.012
		C4 Per capita net income of rural residents	Yuan	Positive	0.052
		C5 Income ratio of urban and rural residents	%	Negative	0.040
		C6 Technical market turnover	Billion yuan	Positive	0.034
		C7 Urban unemployment rate	%	Negative	0.019
	Social life D 0.201	D1 Number of physicians per 10,000 people	Person	Positive	0.042
		D2 Per capita housing area of rural residents	Km^2^/person	Positive	0.030
		D3 Per capita housing area of urban residents	Km^2^/person	Positive	0.021
		D4 Amount of public transportation per 10,000 people in city	-	Positive	0.026
		D5 Number of public libraries	-	Positive	0.028
		D6 Number of college students in 10,000 persons	Person	Positive	0.022
		D7 Per capita urban road area	m^3^	Positive	0.032

**Table 2:** Variable definitions.

Variable	Definitions
*t*	Year of policy issued
*U_t_*	Ecological civilization construction index in year *t*
*p_t_*	Total effectiveness of policy power in year *t*
*i*	Lag period
*PU_t_*	Index of ecological civilization construction of the previous year in year *t*
*C_t_*	Model constant
*f_t_*	Total effectiveness of policy feedback in year *t*
*ε_t_*	Influence of random factors on the dependent variable
*α*, *β_a_*	(*a* = 1, 2, 3, 4) are the coefficients of each variable
*m_t_^φ^*	(*φ* = 1, 2, 3, 4) are the total effectiveness values of policy objectives (resource utilization, environmental protection, economic development, and social life) in year *t*, respectively
*c_t_^δ^*	(*δ* = 1, 2, 3) are the total effectiveness of policy measures (command-control policy, economic-incentive policy and information participation policy) in year *t*, respectively

**Table 3:** Descriptive statistics of the ecological civilization construction index of Jiangsu Province.

Time	Items	Resource Utilization	Environmental Protection	Economic Development	Social Life	Compound Ecological Civilization Construction
2004–2019	Min	0.0103	0.0129	0.0126	0.0085	0.0114
	Max	0.0176	0.0164	0.0315	0.0166	0.0198
	Range	0.0072	0.0035	0.0189	0.0080	0.0084
	Means	0.0140	0.0149	0.0211	0.0126	0.0163
	Total	0.2235	0.2386	0.3368	0.2011	0.2608

**Table 4:** Analysis of the effectiveness of ecological civilization policies from 2004 to 2019.

Variable	Resource Utilization					Environmental Protection				
	Lag	Effect	Coef.	Prob.	VIF	Lag	Effect	Coef.	Prob.	VIF
*C*			−0.4333	0.5831				−0.9670	0.3398	
*PU_t_*		Y	0.8995	0.0026	4.9815		Y	0.7733	0.0116	3.3544
*p*	2	-	0.0108	0.4467	1.6846	2	Y	0.0467	0.0259	3.6632
*m*	0	-	−0.0263	0.1526	3.2329	2	N	−0.0363	0.0410	4.1658
*c* ^1^	1	N	−0.0451	0.0977	6.3915	0	N	−0.0509	0.0245	4.6206
*c* ^2^	0	Y	0.0654	0.0150	2.9068	1	-	0.0083	0.5283	1.9487
*c* ^3^	3	N	−0.0249	0.0873	1.8355	1	-	0.0034	0.7797	2.5172
*f*	1	Y	0.0461	0.0391	3.5092	0	Y	0.0580	0.0079	3.3188
**Index**	R^2^ = 0.969442, AIC = −4.066531, SC = −3.718870, F-statistic = 22.66041, Prob (F-statistic) = 0.001678, D-Watson = 2.975826					R^2^ = 0.916129, AIC = −4.155992, SC = −3.790816, F-statistic = 9.362575, Prob (F-statistic) = 0.007245, D-Watson = 2.289569				
**Variable**	**Economic Development**					**Social Life**				
	**Lag**	**Effect**	**Coef.**	**Prob.**	**VIF**	**Lag**	**Effect**	**Coef.**	**Prob.**	**VIF**
*C*			−0.0195	0.8311				0.0111	0.9568	
*PU_t_*		Y	0.9882	0.0000	4.0949		Y	0.9923	0.0000	3.4380
*p*	1	-	0.0144	0.1461	7.7406	2	Y	0.0106	0.0729	1.3625
*m*	0	Y	0.0431	0.0177	9.6887	0	-	−0.0097	0.2654	4.4460
*c* ^1^	1	N	−0.0143	0.0950	7.2890	1	N	−0.0120	0.0724	2.3262
*c* ^2^	0	N	−0.0185	0.0583	8.3547	0	Y	0.0179	0.0452	2.6290
*c* ^3^	0	-	−0.0074	0.1144	2.6024	3	-	0.0030	0.4820	1.3713
*f*	2	-	0.0037	0.4269	3.6967	0	-	−0.0084	0.4273	5.5995
**Index**	R^2^ = 0.999473, AIC = −6.407371, SC = −6.042195, F-statistic = 1624.357, Prob (F-statistic) = 0.000000, D-Watson = 2.040286					R^2^ = 0.997478, AIC = −5.924984, SC = −5.577323, F-statistic = 282.5101, Prob (F-statistic) = 0.000003, D-Watson = 2.580630				

Note: Significance is 10%; “Y” means that it promotes the construction of ecological civilization, “N” means that it has a negative effect on the construction of ecological civilization, and “-” means that it has no significant influence on the construction of ecological civilization.

## Data Availability

Data are available under request to the corresponding author.

## Supplementary Materials

The following Supporting Information can be downloaded at: https://www.mdpi.com/article/10.3390/ijerph19010388/s1, Table S1: List of Ecological Civilization Construction Policies of Jiangsu Province from 2004 to 2019.

## Appendix A

**Table A1:** Policy Quantification Standards.

Items		Criteria
**Policy Power**		
-	5	Law promulgated by the Jiangsu Provincial People’s Congress and its Standing Committee
	4	Regulations, directives and orders issued by Jiangsu Provincial People’s Government
	3	Provisional regulations and provisions, plans, decisions, opinions, measures and standards promulgated by the Jiangsu Provincial People’s Government; regulations, provisions and decisions promulgated by various departments
	2	Opinions, measures, programs, guidelines, provisional provisions, rules, conditions and standards promulgated by various ministries and commissions
	1	Notices, announcements, planning
**Policy objectives**		
Environmental protection	5	Taking environmental protection as one of the most important objectives; clearly setting out different targets for different periods of environmental protection; clearly pointing out targets such as emission reduction, environmental quality, and environmental infrastructure; and making comprehensive requirements in terms of legislation, publicity, and implementation
	3	Taking economic development as one of the important goals, lack of quantitative indicators and specific practices
	1	Only macroscopically expressing the vision and expectations of environmental protection civilization
Economic development	5	Taking economic development as one of the most important objectives; the specific targets of different periods of ecological economic development are clearly defined; the target indicators such as industrial green level, energy structure optimization and green economic development are clearly pointed out; and all requirements are provided in terms of legislation, publicity and implementation
	3	Taking economic development as one of the most important goals, lack of quantitative indicators and specific practices
	1	Only macroscopically expressing the vision and expectation of economic development
Resource utilization	5	Taking resource utilization as one of the most important objectives; clarify the different targets for different periods of resource utilization; clearly indicate target indicators such as energy utilization rate and pollutant reuse rate; and comprehensively request legislation, publicity, and implementation.
	3	Taking resource utilization as one of the important goals, lack of quantitative indicators and specific practices
	1	Only macroscopically expressing the vision and expectations of the use of civilization
Social life	5	Taking social life as one of the most important goals; clarifying the different specific goals of different periods of social life; clearly indicating the target indicators of residents’ living standards and cultural quality; and making comprehensive requirements from legislation, publicity, and implementation.
	3	Taking social life as one of the important goals, lacking quantitative indicators and specific practices
	1	Only macroscopically expressing the vision and expectations of social life civilization
**Policy measures**		
Command- control policy	5	Formulated measures and standards for the implementation of mandatory ecological civilization construction; formulated measures for assessment, inspection, supervision and inspection of ecological civilization; formulated specific litigation mechanisms; and formulated mandatory management measures specifically for promoting the development of ecological civilization
	3	Clearly request the formulation of assessment methods for ecological civilization construction; explicitly require the implementation of ecological civilization construction assessment, supervision and inspection; require the formulation of safeguard mechanisms; explicitly require the formulation of relevant policies or systems to promote the construction of ecological civilization
	1	Only mention or refer to the terms of Command-Control Policy Tool 5 and 3 points of the evaluation criteria
Economic incentive policy	5	Strong support was given to budgets, subsidies, subsidies, interest subsidies, and incentives, and financial subsidies, subsidies, inputs, incentives, or support methods were proposed; for green economic transformation, improvement of environmental quality, and development of green technology internships. Economic incentive schemes
	3	Clearly proposed to support green economic transformation and the development of green technology in the financial aspect, but did not propose relevant support quotas, formulate relevant methods or catalogues; insisted on improving environmental quality, but did not formulate specific economic penalties and incentives for destroying or protecting environmental behaviors
	1	Only mention or relate to the terms of the 5 and 3 points of the economic incentive policy tool
Information participation policy	5	Vigorously advocate ecological life, cultivate ecological culture, and formulate specific implementation and promotion methods or programs for publicity in light of realistic characteristics; and formulate detailed guidance systems and measures for ecological lifestyle and ecological culture cultivation methods.
	3	It is clearly stated that it is necessary to advocate ecological life, cultivate ecological culture, require propaganda and promotion, and formulate a series of macro measures. However, no specific implementation measures and plans have been formulated.
	1	Only mention or involve the participation of the information in the policy tool 5 points and 3 points in the evaluation criteria
**Policy feedback**		
-	5	There are detailed monitoring methods and clear responsible departments, with regular feedback documents and corresponding accountability mechanisms.
	3	Have a clear supervision and responsible department, but insufficient feedback
	1	Only mention supervision and feedback, and there is no specific department or method
