# Supplementary material for: Evaluating the Effectiveness of Regional Ecological Civilization Policy: Evidence from Jiangsu Province, China
Source: Int J Environ Res Public Health. 2021 Dec 30;19(1):388. doi: 10.3390/ijerph19010388 (PMC8744889; doi:10.3390/ijerph19010388)
Supplement: Supplementary file 1 [file ijerph-19-00388-s001.zip › ijerph-1510030-supplementary.pdf]

Table S1: List of Ecological Civilization Construction Policies of Jiangsu Province from 2004 to 2019

Years	Publishing department	Names of policy documents	File number
2004.12.28	Jiangsu Provincial People's Government	Notice of the Jiangsu Provincial People's Government on Printing and Distributing the Outline of the Construction Plan of Jiangsu Ecological Province	SZF [2004] No.6
2005.09.15	General Office of the Jiangsu Provincial People's Government	Notice of the General Office of the Provincial Government on Printing and Distributing the Decompression Table of Key Construction Projects of Ecological Province	SZBF [2005] No.103
2006.06.23	General Office of the Jiangsu Provincial People's Government	Notice on Forwarding Provincial Financial Stability Work Coordination Group's Guidance on the Construction of Financial Ecological Environment in the Province	SZBF [2006] No.50
2007.01.12	General Office of the Jiangsu Provincial People's Government	Notice of the General Office of the Provincial Government on Studying and Formulating the Main Tasks and Key Projects for the Construction of the Ecological Province in 2007	SZBF [2007] No.3
2007.08.14	General Office of the Jiangsu Provincial People's Government	Notice of the Provincial Government Office to Forward the Implementation Opinions of the Provincial Financial Stability Work Coordination Group Office on the Construction of Financial Ecological County in Jiangsu Province	SZBF [2007] No.100
2008.02.03	General Office of the Jiangsu Provincial People's Government	Notice of the General Office of the Provincial Government on the Release of the Provincial Ecological Public Welfare Forest Area Index	SZBF [2008] No.6
2008.04.28	CPC Jiangsu Provincial Committee, Jiangsu Provincial People's Government	Notice of the General Office of the People's Government of Jiangsu Province on Printing and Distributing the "Eleventh Five-Year Plan for Environmental Protection and Ecological Construction of Jiangsu Province"	SZBF [2008] No.26
2009.04.09	Education Department of Jiangsu	Notice of the Jiangsu Provincial Greening Committee, the Jiangsu Provincial Department of Education, and the Jiangsu Provincial Forestry Bureau on Jointly Launching the Activities of "Promoting Ecological Civilization and Building a Green Campus Together"	SLW [2009] No.2
2009.06.29	Jiangsu Forestry Bureau	Notice of the Jiangsu Provincial Forestry Bureau, the Jiangsu Provincial Department of Education, and the Jiangsu Provincial Committee of the Communist Youth League on the Establishment of the "Jiangsu Ecological Civilization Education Base"	SLF [2009] No.9
2009.06.29	Jiangsu Forestry Bureau	Notice of the Jiangsu Provincial Forestry Bureau, the Jiangsu Provincial Department of Education, and the Jiangsu Provincial Committee of the Communist Youth League on Printing and Distributing the Administrative Measures for the Ecological Civilization Education Base of Jiangsu Province	SLF [2009] No.10
2010.11.18	Jiangsu Provincial People's Government	"Opinions of the Jiangsu Provincial Committee of the Communist Party of China and the People's Government of Jiangsu Province on Accelerating the Construction of the Ecological Province and Improving the Level of Ecological Civilization in an All-round Way"	SF [2010] No.24
2011.04.22	Jiangsu Higher People's Court	Opinions of the Jiangsu Higher People's Court on Providing Judicial Protection for Accelerating the Construction of Ecological Province and Promoting the Level of Ecological Civilization	SGF [2011] No.201
2011.08.30	Jiangsu Provincial People's Government	Notice of the CPC Jiangsu Provincial Committee and the Jiangsu Provincial People's Government on Printing and Distributing the Action Plan for Promoting Ecological Civilization Construction Project	SF [2011] No.26
2012.03.13	Jiangsu Provincial Department of Land and Resources	Notice of the Jiangsu Provincial Department of Land and Resources on the Tasks for the Construction of Ecological Civilization Construction Projects	SGTZF [2012] No.104

		(Abandoned Mine Geological Environment Management) in 2011-2015	
2012.04.01	Jiangsu Provincial Department of Environmental Protection	Notice on Carrying out the 2011 Eco-Civilization and Eco-Civil Envoys Appraisal and Recognition Activities	SHB [2012] No.70
2012.04.17	Jiangsu Provincial People's Government	Notice of the Provincial Government on Printing and Distributing the "Twelfth Five-Year Plan" Environmental Protection and Ecological Construction Plan of Jiangsu Province	SZF [2012] No.51
2013.02.25	Jiangsu Provincial People's Government	Several Opinions of the Provincial People's Government on Strengthening Environmental Protection and Promoting the Construction of Ecological Civilization	SZF [2013] No.11
2013.04.02	General Office of the Jiangsu Provincial People's Government	Notice of the General Office of the Provincial Government on Printing and Distributing the Division of Labor of the Key Work Departments of the “Twelfth Five-Year Plan” for Environmental Protection and Ecological Construction in Jiangsu Province	SZBF [2013] No.43
2013.04.19	Jiangsu Provincial Department of Environmental Protection	Notice on Hosting the Jiangsu Province Green School Ecological Civilization Performance Competition	SHB [2013] No.106
2013.06.14	Jiangsu Provincial Department of Water Resources	Provincial Water Resources Department's Opinions on Promoting the Construction of Water Ecological Civilization	SSZ [2013] No.26
2013.06.18	Jiangsu Provincial Department of Water Resources	Notice of Jiangsu Provincial Department of Water Resources on Launching Pilot Work of Water Ecological Civilization Construction in the Province	SSZ [2013] No.30
2013.07.20	Jiangsu Provincial People's Government	Notice of the Provincial Government on Printing and Distributing the Planning of Ecological Civilization Construction in Jiangsu Province (2013-2022)	SZF [2013] No.86
2013.07.21	CPC Jiangsu Provincial Committee, Jiangsu Provincial People's Government	Opinions of the CPC Jiangsu Provincial Committee and the Jiangsu Provincial People's Government on Deeply Advancing the Construction of Ecological Civilization and Taking the Lead in Building the National Ecological Civilization Construction Demonstration Zone	SF [2013] No.11
2013.08.09	Jiangsu Provincial Department of Environmental Protection	Notice on Printing and Distributing the Evaluation Standards of Ecological Civilization Education Base in Jiangsu Province	SHB [2013] No.217
2013.08.09	Jiangsu Provincial Department of Environmental Protection	Notice on Printing and Distributing the Administrative Measures for the Ecological Civilization Education Base of Jiangsu Province	SHB [2013] No.218
2013.09.23	Jiangsu Provincial People's Government	Notice of the Provincial Government on Printing and Distributing the Regional Protection Plan for Ecological Red Lines in Jiangsu Province	SZF [2013] No.113
2013.09.25	Jiangsu Provincial Department of Water Resources	Notice of Jiangsu Provincial Department of Water Resources on Piloting the Construction of Water-Ecological Civilized City in the Province	SSZ [2013] No.48
2013.12.26	General Office of the Jiangsu Provincial People's Government	Circular of the General Office of the Provincial Government on Forwarding the Interim Measures for the Transfer of Ecological Compensation Transfers of Jiangsu Provincial Department of Environmental Protection	SZBF [2013] No.193
2014.03.13	General Office of the Jiangsu Provincial People's Government	Notice of the General Office of the Provincial Government on Forwarding the Interim Measures for the Provincial Environmental Protection Supervision and Management Assessment of the Provincial Red Department of the Provincial Department of Environmental Protection	SZBF [2014] No.23
2015.07.06	Jiangsu Forestry Bureau	Notice on Printing and Distributing the "Jiangsu Province Ecological Protection and Construction Plan (2014.2020)"	SFGNJF [2015] No.667
2015.10.20	Jiangsu Provincial People's Government	"Implementation Opinions of Jiangsu Provincial Party Committee and Provincial Government on	SF [2015]

		Accelerating the Construction of Ecological Civilization"	No.30
2016.07.01	General Office of the CPC Jiangsu Provincial Committee; General Office of the Jiangsu Provincial People's Government	"Implementation Rules for the Investigation of Responsibility for Ecological Environment Damage of Leading Cadres of the Party and Government in Jiangsu Province"	SBF [2016] No.10
2016.08.22	General Office of the CPC Jiangsu Provincial Committee	Notice of the Jiangsu Provincial People's Government of the Jiangsu Provincial Committee of the Communist Party of China on Printing and Distributing the "Provisions on the Responsibility of Ecological Environment Protection in Jiangsu Province (Trial)"	SF [2016] No.37
2016.12.06	Jiangsu Provincial People's Government	Notice of the Provincial Government on Printing and Distributing the Implementation Plan for the Pilot Work of the Reform of the Compensation System for Ecological Environment Damage in Jiangsu Province	SZF [2016] No.159
2017.01.19	Jiangsu Provincial People's Government	Notice of the Provincial Government on Printing and Distributing the Implementation Plan for the Construction of the Ecological Protection Network in the Subei Area of Northern Jiangsu	SZF [2017] No.7
2017.05.15	General Office of the Jiangsu Provincial People's Government	Guiding Opinions of the General Office of the Provincial Government on Promoting the Construction of Ecological Protection Leading Areas and Ecological Protection Special Zones	SZBF [2017] No.73
2017.06.20	General Office of the Jiangsu Provincial People's Government	Notice of the General Office of the Provincial Government on Printing and Distributing the Implementation Plan for the Construction of the Ecological Environment Monitoring Network of Jiangsu Province	SZBF [2017] No.91
2017.08.25	General Office of the Jiangsu Provincial People's Government	Notice on Printing and Distributing the Management Regulations and Indicators of Model City and County (City, District) for the Construction of Ecological Civilization in Jiangsu Province	SHB [2017] No.259
2017.08.25	General Office of the Jiangsu Provincial People's Government	Notice on Printing and Distributing Model Townships (Streets) and Village Management Regulations and Indicators for Ecological Civilization Construction in Jiangsu Province	SHB [2017] No.260
2017.10.09	Jiangsu Provincial People's Government	Notice of the Provincial Government on Printing and Distributing the Ecological River and Lake Action Plan of Jiangsu Province (2017-2020)	SZF [2017] No.130
2017.12.24	General Office of the Jiangsu Provincial People's Government	Opinions of the General Office of the Provincial Government on Accelerating the Construction of Ecological Agriculture in the Taihu Basin	SZBF [2017] No.153
2018.06.09	Jiangsu Provincial People's Government	Notice of Provincial Government on Printing and Distributing the National Protection Plan for Ecological Red Lines in Jiangsu Province	SZF [2018] No.74
2018.08.30	CPC Jiangsu Provincial Committee, Jiangsu Provincial People's Government	Notice of the General Office of the CPC Jiangsu Provincial Committee and the General Office of the Jiangsu Provincial Government on Printing and Distributing "the Implementation Plan for the Reform of the Compensation System for Ecological Environment Damage in Jiangsu Province"	SBF [2018] No.38
2018.10.07	General Office of the CPC Jiangsu Provincial Committee	Implementation Opinions of the CPC Jiangsu Provincial Committee and the Jiangsu Provincial People's Government on Comprehensively Strengthening Ecological Environmental Protection and Resolutely Fighting Pollution Prevention and Control	SF [2018] No.24
2018.12.18	General Office of the Jiangsu Provincial People's Government	Notice of the General Office of the Provincial Government on Printing and Distributing the Implementation Plan for the Protection of Urban Centralized Drinking Water Sources in Jiangsu Province	SZBF [2018] No.107
2019.01.15	General Office of the Jiangsu Provincial People's Government	Notice of the General Office of the Provincial Government on Printing and Distributing the Implementation Plan for the Battle of Taihu Lake Governance in Jiangsu Province	SZBF [2019] No.4

2019.01.17	General Office of the Jiangsu Provincial People's Government	Implementation Opinions of the General Office of the Provincial Government on Strengthening the Protection of Aquatic Organisms of the Yangtze River in the Jiangsu Section	SZBF [2019] No.7
2019.03.06	General Office of the Jiangsu Provincial People's Government	Notice of the General Office of the Provincial Government on Printing and Distributing the Implementation Plan for the Construction of Ecological Environmental Standards System in Jiangsu Province (2018-2022)	SZBF [2019] No.26
2019.03.07	General Office of the Jiangsu Provincial People's Government	Notice of the General Office of the Provincial Government on Printing and Distributing the Three-Year Environmental Infrastructure Construction Plan in Jiangsu Province (2018-2020)	SZBF [2019] No.25
2019.03.07	General Office of the Jiangsu Provincial People's Government	Notice of the General Office of the Provincial Government on Printing and Distributing the Three-year Construction Plan for Ecological Environment Monitoring and Monitoring System in Jiangsu Province (2018-2020)	SZBF [2019] No.27
2019.06.02	General Office of the Jiangsu Provincial People's Government	Notice of the General Office of the Provincial Government on Printing and Distributing the Implementation Plan of the Action Plan for the Protection and Restoration of the Yangtze River in Jiangsu Province	SZBF [2019] No.52
2019.08.31	Jiangsu Provincial People's Government	Implementation Opinions of the General Office of the Jiangsu Provincial Government on Strengthening the Ecological Protection and Scientific Utilization of Hongze Lake	SZBF [2019] No.72
2019.12.20	General Office of the Jiangsu Provincial People's Government	Notice of the General Office of the Provincial Government on Printing and Distributing the General Work Plan for the Unified Confirmation and Registration of Natural Resources in Jiangsu Province	SZBF [2019] No.87
